# The Role of Interaction between Mitochondria and the Extracellular Matrix in the Development of Idiopathic Pulmonary Fibrosis

**DOI:** 10.1155/2021/9932442

**Published:** 2021-10-18

**Authors:** Kamil Siekacz, Wojciech J. Piotrowski, Mikołaj A. Iwański, Paweł Górski, Adam J. Białas

**Affiliations:** ^1^Department of Pathobiology of Respiratory Diseases, 1st Chair of Internal Medicine, Medical University of Lodz, Poland; ^2^Department of Pneumology and Allergy, 1st Chair of Internal Medicine, Medical University of Lodz, Poland

## Abstract

Idiopathic pulmonary fibrosis (IPF) is a condition which affects mainly older adults, that suggests mitochondrial dysfunction and oxidative stress, which follow cells senescence, and might contribute to the disease onset. We have assumed pathogenesis associated with crosstalk between the extracellular matrix (ECM) and mitochondria, mainly based on mitochondrial equilibrium impairment consisting of (1) tyrosine kinases and serine-threonine kinase (TKs and ST-Ks) activation via cytokines, (2) mitochondrial electron transport chain dysfunction and in consequence electrons leak with lower ATP synthesis, (3) the activation of latent TGF-*β* via *α*V*β*6 integrin, (4) tensions transduction via *α*2*β*1 integrin, (5) inefficient mitophagy, and (6) stress inhibited biogenesis. Mitochondria dysfunction influences ECM composition and vice versa. Damaged mitochondria release mitochondrial reactive oxygen species (mtROS) and the mitochondrial DNA (mtDNA) to the microenvironment. Therefore, airway epithelial cells (AECs) undergo transition and secrete cytokines. Described factors initiate an inflammatory process with immunological enhancement. In consequence, local fibroblasts exposed to harmful conditions transform into myofibroblasts, produce ECM, and induce progression of fibrosis. In our review, we summarize numerous aspects of mitochondrial pathobiology, which seem to be involved in the pathogenesis of lung fibrosis. In addition, an increasing body of evidence suggests considering crosstalk between the ECM and mitochondria in this context. Moreover, mitochondria and ECM seem to be important players in the antifibrotic treatment of IPF.

## 1. Introduction

Idiopathic Pulmonary Fibrosis (IPF) is a chronic, irreversible, and lethal lung disease of unknown origin [1]. This most common type of idiopathic interstitial pneumonia (IIP) affects an increasing number of adults over 50 years of age and is becoming a significant health problem worldwide. Each year in Europe, 40,000 new cases are registered [2, 3]. Although patients' median survival time before the introduction of antifibrotic drugs was estimated to be between 3 and 5 years after initial diagnosis, the true range strongly depends on the phenotype of disease [4], and the disease course is generally variable and unpredictable. IPF is limited to the lungs and is defined by the radiological pattern of usual interstitial pneumonia (UIP) which, in short, is characterized by a subpleural, basal predominance, the presence of reticular abnormalities, and honeycombing with or without traction bronchiectasis [5]. The primary features of the clinical presentation of IPF include insidious onset of dyspnoea (90% of cases), fatigue (69%), loss of appetite (67%), dry coughing (53%), and dyspnoea with upper airway infection (32%). The length of time from onset of symptoms to diagnosis is on average 21.8 months. Thus far, only antifibrotic therapy with pirfenidone/nintedanib and lung transplantation has been proven to prolong survival. Therefore, identifying early biomarkers of illness and finding a novel form of treatment consistent with disease biology are necessary [6].

A favored hypothesis of IPF pathobiology focuses on local microinjuries in the alveolar epithelial layer of the lung. Microdamages are caused by exposure to harmful environmental factors including tobacco smoke, gastroesophageal reflux microbial, and viral agents [7]. These events lead to alveolar epithelial dysfunction and induce senescence of AEC [8]. An acquired cellular phenotype initiates pulmonary fibrosis through an abnormal secretory pattern and an increased resistance to apoptosis of myofibroblasts [7]. Senescent epithelial cells (SECs) overproduce and secrete cytokines, chemoattractants, and growth factors [9]. Profibrotic mediators act on resident fibroblasts via the tyrosine kinase and serine–threonine kinase pathways and initiate fibroblast proliferation and myofibroblast differentiation [10]. Overstimulated fibroblasts and myofibroblasts create clusters of cells called fibroblastic foci. These cells synthesize collagen, elastin, matrix metalloproteinases (MMPs), lysyl oxidase-like 2 (LOXL2), and lysophosphatidic acid (LPA) altering the composition and biomechanics of the ECM [11]. The process of lung fibrosis leads to the destruction of lung architecture which disrupts blood flow and diffusion of gases [1, 12]. The malfunction of cellular respiration and regeneration processes accelerates the progression of the disease [13]. Therefore, ageing and several other hallmarks of this process including genomic instability, shortened telomeres, oxidative injury, proteostatic dysregulation, and endoplasmic reticulum stress are linked with IPF [8]. Mitochondria have been identified as playing a significant role in the pathophysiology of the aforementioned processes [14].

## 2. Mitochondrial Processes Involved in IPF

Mitochondria are not isolated static structures as they create dynamic spatial reticular cluster networks in a cell known as a chondriome [15]. The shape, branching, morphology, and localization of clusters depend on the cell type, their actual metabolism, energy requirement, cell cycle phase, cytosol toxins, and other stressors [16, 17].

Mitochondrial units are capable of fusion and division within the cluster. Fusion leads to the formation of long reticular filaments that spread over the entire cell. The network is a branched cylindrical-shaped complex organelle composed of anywhere from a hundred to a thousand mitochondrial units [18].

On the other hand, when cells need to proliferate and divide, complex clusters of mitochondria also have to undergo fission to distribute smaller clusters into higher energy demand regions of the cell [19]. The balance between fission and fusion is one of the compensatory mechanisms of mitochondrial damage [20]. In an emergency, an impaired mitochondrion will fuse with another to mitigate dysfunction through the exchange of compounds crucial for proper functioning This type of crosscomplementation maximizes oxidative capacity in response to stress [16].

### 2.1. Mitophagy

**Figure 1:**
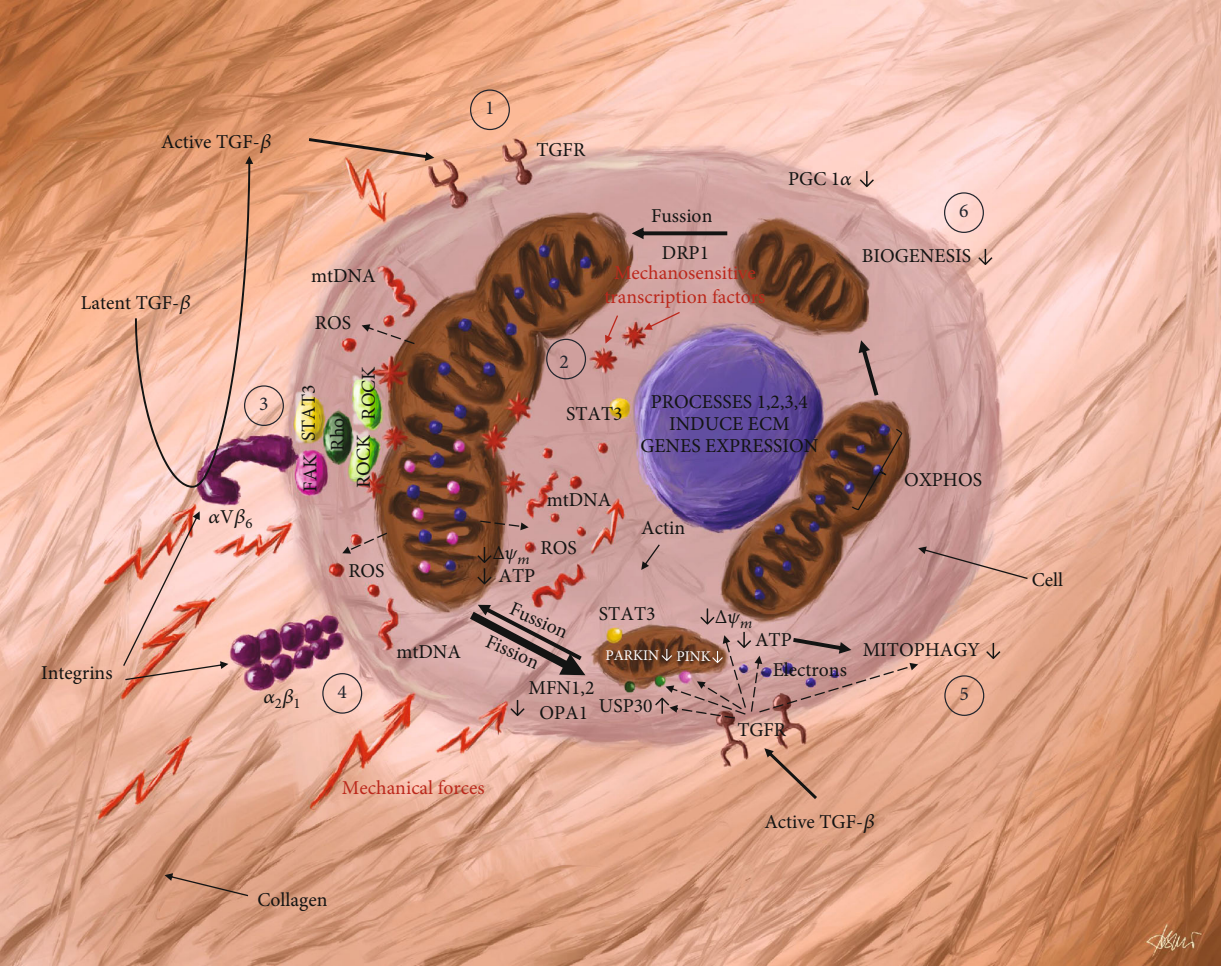
Extracellular matrix-induced mitochondrial imbalance of IPF pathobiology in a cell. Mitochondrial equilibrium impairment is induced by several processes including subsections: (1) Activation of TKs and TS-Ks via cytokines. (2) Mitochondrial ETC, OXPHOS dysfunction, and in consequence electrons leak with lower ATP synthesis, increased mtROS production, mtDNA damage, and release to mitochondria environment. (3) Transformation of latent TGF to active TGF and activation of pathways: FAK-Rho-ROCK, FAK-STAT3 via *α*V*β*6 integrin, (4) tension transduction via *α*2*β*1 integrin that promotes mitochondria dysfunction, (5) inefficient mitophagy, and (6) stress inhibited biogenesis. Moreover, processes 1,2,3, and 4 activate ECM gene expression responsible for fibrosis. Abbreviations: TKs and TS-Ks: tyrosine kinase and serine–threonine kinases; ECM: extracellular matrix; ETC: electron transport chain; OXPHOS: mitochondria oxidative phosphorylation system; ATP: adenosine triphosphate; mtDNA: mitochondrial DNA; mtROS: mitochondrial reactive oxygen species; lTGF: latent tumor growth factor; aTGF: active tumor growth factor; FAK: focal adhesion kinase; ROCK: Rho protein kinase, STAT3: signal transducer and activator of transcription 3; PINK1: PTEN-induced kinase 1; USP: ubiquitin carboxyl-terminal hydrolase; PGC1*α*: PPAR*γ* coactivator-1*α*; *ΔΨ*m: mitochondrial membranes potential.

Conversely, to maintain a healthy mitochondrial network, cells require an efficient mechanism of mitochondrial elimination. Therefore, in case of severe mitochondrial stress, impaired mitochondrial units of the network are selected, separated, and targeted for elimination by the mitochondrial quality control (MQC) [21]. The MQC is provided by mitophagy which is a type of autophagy responsible for the recognition and elimination of defective mitochondrion [22]. This process of cellular component degradation prevents the accumulation of swollen and defective mitochondria. The depolarization status of mitochondrion was indicated as a major selection signal for mitophagy [23]. This phenomenon is regulated mainly by the PTEN-induced kinase 1 (PINK1)-Parkin pathway. In healthy cells, PINK1 is anchored to the outer mitochondrial membrane (OMM) through which exposing its N-terminal domain to the matrix in a manner-dependent on the mitochondrial membrane potential (MMP). The properly functioning mitochondrion has a stable MMP and therefore, mitochondria processing peptidase (MPP) and the intramembrane serine protease (PARL) can degrade PINK1 units [23]. Conversely, in dysfunctional mitochondria, the MMP is changed that impairs degradation and consequently, PARK1 units dimerize and crossphosphorylate each other to acquire phosphorylation activity. In this stage, PINK1 phosphorylates parkin and ubiquitin. The triggered cascade of ubiquitination drives mitochondria to mitophagy [22]. This process is modulated by deubiquitinating enzymes (DUBs) which remove the ubiquitin tags added by Parkin and inhibit mitophagy [24]. The decreased level of PINK, Parkin, and in consequence deficiency of mitophagy has been confirmed in the lung cells of IPF patients (Figure 1, Subsection 5).

Excessive ECM protein production and deposition induce mechanical forces. Mechanical stress affects the mitochondrial cycle via the integrin's cytoskeleton complex. ECM stimulates cytokine production. Therefore, impaired mitophagy and mitochondria biogenesis promote IPF progression [25]. (Figure 1. Subsections 5 and 6.) The dysregulation of these mechanisms has been associated with IPF and could be a potential target of disease therapy [26]. Particular attention should be focused on highly selective inhibitors of ubiquitin-specific protease 30 (USP30) which accelerate mitophagy [24]. USP30 inhibition seems to be a potential therapeutic target that could augment mitochondrial ubiquitination and enhance mitophagy [27]. Pharmacological modulation of MQC that targets biogenesis, mitophagy, and fission/fusion could lead to the prevention of mitochondrial dysfunction and ultimately, fibrosis [22, 28].

### 2.2. Role of Mitochondrial Equilibrium in Pulmonary Fibrosis

Oxidative stress plays a crucial role in the pathogenesis of IPF. Increased ROS production (H_2_O_2_, OH^.-^, O^.-^) and antioxidant reduction (glutathione, catalase, superoxide dismutase) lead to (1) cytokines and growth factor activation which initiate the development of the inflammatory process, (2) disorganization of ECM and extra-intracellular tension formation; (3) loss of AECs by nuclear DNA, mtDNA damage, and increased telomere shortening; (4) induction of cellular senescence; and (5) endoplasmic stress formation [8, 29, 30]. Oxidative stress is the outcome of mitochondrial dysfunction. Mitochondrial alterations are associated with ageing. Moreover, mitochondrial impairment increases gradually with age. Indeed, lung cells of patients with IPF are more prone to cellular stress related to mitochondrial dysfunction and consequently, fibrosis [31].

Additionally, exposure to harmful environmental factors, especially smoke and acids, propagates free radical formation and, consequently, oxidative damage of lipids, proteins, and DNA [32]. MtDNA is more susceptible to oxidative stress than nuclear genetic material. Progressive damage of mtDNA contributes to mitochondrial dysfunction. This phenomenon triggers a vicious cycle of ROS formation with mtDNA damaging. MtDNA is being released from cells to surrounding lung tissue (Figure 1.  Subsection 2). Therefore, mtDNA is directly involved in the pathophysiology of IPF [33, 34]. It was proven that altering mtDNA could lead to defects in the mitochondria oxidative phosphorylation system (OXPHOS) which would decrease ATP production and increase the yield of mitochondrial reactive oxygen species (mROS) [22]. Plasma mtDNA can be utilized to predict mortality and response to treatment in IPF patients [35], that was implemented using the threshold of copy number of mitochondrially encoded ATP synthase membrane subunit 6 (MT-ATP6) [36]. Otherwise, to stop oxidative stress escalation, dysfunctional mitochondria have to be eliminated or regenerated [16].

## 3. Crosstalk between the ECM and Mitochondria in IPF

### 3.1. Influence of Mechanical Tension on Fibroblast to Myofibroblast Transformation and Excessive ECM Production

The pathogenesis of IPF focuses on the dysfunctional epithelium, fibrogenesis, and lung fibrosis as the final effect. Microinjuries and mechanical and oxidative stress activate fibroblasts and promote their proliferation and differentiation into myofibroblasts. The myofibroblasts are the main cells responsible for excessive ECM protein production [1]. ECM expansion decreases the alveolar epithelial cell (AEC) population. A microenvironment with a smaller amount of cells, improper basement membrane contacts, and enhanced ECM interaction sites induces myofibroblast proliferation. Moreover, overstimulation by platelet-derived growth factor (PDGF), epithelial growth factor (EGF), and fibroblast growth factor (FGF) promotes this process [13].

### 3.2. Lung Tissue Morphology and Biomechanics in IPF

Matrisome becomes abundant in collagen I, periostin 1, osteopontin, fibronectin, and fibulin 1c, thus replacing agrin laminins and collagen IV production [37]. ECM glycosaminoglycan distribution and deposition of lysyl oxidase-like 2 (LOXL2) and the lysophosphatidic acid (LPA) pattern stabilize the matrix and significantly stiffen local lung tissue [7, 38]. Therefore, a comparison of the median value of Young's modulus, which defines the relationship between stress (force per unit area) and strain (proportional deformation), shows that it was 10-fold higher in the tissue of IPF patients than in healthy volunteers [28]. The ECM remodeling in the lung parenchyma is the reason for shortness of breath, the first clinical symptom of disease onset. Moreover, forced vital capacity (FVC) decline, as an indirect measurement of lung rigidity, correlates with mortality and is a standard in monitoring disease progression [38]. Small areas from 1.3 × 10^4^ to 9.9 × 10^7^ *μ*m of active fibrosis (called fibroblast foci) drive fibrotic zone formation. Those independent structures arising in response to microdamage and repair impairment lead to the characteristic morphology of interstitial pneumonia which includes subpleural and paraseptal fibrosis with a honeycombing pattern in histopathology [39]. Consistently, HRCT reveals reticular opacities associated with traction bronchiectasis. Scars are formed in the lower lobes of the lung, and this is seen as a rare ground-glass opacification in HRCT imaging [40]. The imbalance between synthesis, deposition, degradation, and clearance of the ECM leads to chronic and progressive fibrotic and degenerative remodeling processes. The lung tissue biomechanic alteration influences cells via intracellular organelle dysfunction [37, 38].

### 3.3. Cytoskeleton Tensions

Mechanical stress is absorbed by the cell cortex that consists of actin and actomyosin fibres that underlie the surface of the cell membrane. The cell cortex is anchored to the cell membrane by the ERM protein family which includes ezrin, radixin, and moesin. ERM proteins organize membrane domains and interact with transmembrane proteins influencing the cytoskeletal organization. This group of proteins provides membrane-cytoskeletal crosstalk and regulates intracellular pathway activation [41]. Eoesin regulates cortical stiffness by driving Rho1 and myosin II protein localization. The cell cortex is reinforced by crosslinking proteins including spectrin, actin, and ankyrin which together form an array of periodic rings that attach to transmembrane proteins [42, 43]. Mitochondria are embedded into the cytoskeletal framework of the cell, especially interacting with actin filaments. Actin filaments tend to accumulate and transduce pressure onto the mitochondrial surface which initiates constriction and results in the fission of the mitochondrial network [44]. Nonmuscular myosin II (NMII) accumulates spherically around constrictions of the mitochondrial tubular network and changes the properties of local actin condensation. The high surface tension causes mitochondrial dysfunction. A consequence of mechanical tension is induction of oxidative stress, ROS formation, and decreased OXPHOS and ATP production. Mechanical stretch and cellular hypoxia cause endoreticulum stress (ER stress) in AEC of IPF patients [45]. It was indicated that microarchitectural changes and alveolar collapse precede lung fibrosis. Mitochondria are sensitive to cytoarchitectural changes which influence on MMP [46]. Based on the link between mitochondria dysfunction and surface tension enhancement, mechanical forces induce ECM deposition within alveolar septa, that confirms the concept that mitochondrial dysfunction induces IPF progression which is caused by mechanical stress and vice versa [47].

### 3.4. Integrin's Interactions

Mechanical stiffness and tension are transferred directly from the ECM to the cytoskeleton scaffold via integrin's (Figure 1.  Subsection 4) [48]. They play critical roles in receiving information from the microenvironment and transducing it via the cellular membrane (CM) to the matrix of fibroblasts, epithelial, and immune cells. Integrin's activate downstream pathways include intracellular adaptors such as (1) p130Cas and Grb2, (2) cytosolic tyrosine kinases, such as Src family kinase (SFK) and focal adhesion kinase (FAK), (3) growth factor receptors including EGFR and platelet-derived growth factor receptors (PDGF), and (4) cytokine receptors, such as the IL-3 receptor [49]. They serve as tissue integrity adhesion mediators, especially in the lung. Several integrins are associated with IPF [50, 51]. Especially, the upregulated *α*V*β*6 integrin is responsible for the activation of constitutively expressed latent transforming growth factor-*β* (TGF-*β*) (Figure 1.  Subsection 3), the pivotal mediator in the development of pulmonary fibrosis. Moreover, the interaction between the *α*2*β*1 integrin and collagen has two functions that depend on collagen type. In the case of monomeric collagen, it maintains stability via the phosphoinositide 3-kinase (PI3K)–Akt–S6K1 signaling pathway and the proliferative activity of fibroblast cells. In the second type, ECM abundant in polymeric collagen generates enhanced integrin ligand-binding signal. Therefore, increased activity of Akt and S6k1 proteins suppresses the constant activity of PTEN proteins. PTENs are the main inhibitors of the phosphoinositide 3-kinase (PI3K)–Akt–S6K1 signaling pathway and limit excessive fibroproliferation. It seems that this mechanism is aberrant in the fibroblasts of patients with IPF [52].

Additionally, integrins, in response to stress, activate FAK and f-actin polymerization which leads to stress fibre formation [53]. FAK phosphorylation induces activation pathways of Rho kinase (ROCK) through Rho protein complexing. ROCK increases actomyosin contractibility. As a response to the increased tension, mechanosensitive proteins such as yes-associated protein (YAP) and transcriptional coactivator with PDZ-binding motif (TAZ) are activated. Those transcription factors translocate into the nucleus of the cell to induce the transcription of profibrotic genes (Figure 1. Subsections 2 and 3.), that triggers a vicious cycle of ECM protein production and in effect, further microenvironment stiffness which drives fibrosis [38].

### 3.5. Receptors, Pathways, and Second Messengers

Mechanical stress, oxidative stress, cytokines, and receptor tyrosine kinases induce signal transduction and activation of transcription 3 (STAT3) which regulates transcription of antiapoptotic proteins (B-cell lymphoma-extra large, Survivin, cyclin D1 [CCND1]) and transcription factors (c-Myc and Twist-related protein 1). Additionally, the ECM-integrin-FAK signaling pathway directly regulates mitochondrial function through the STAT3 protein. This protein activates and facilitates maintaining optimal oxidative phosphorylation in complexes I, II, and V that provide proper MMP [53]. Therefore, STAT3 suppresses autophagy and preserves malfunctioning mitochondria from degradation by mitophagy [54] (Figure 1. Subsection 5). The level of STAT3 in fibroblasts from patients with IPF correlates with progression of the disease. Small molecular inhibitors directed against STAT3 alleviate fibrosis in the mouse bleomycin model of IPF [55]. There is a strong association between STAT3 activation and oxidative-induced senescence, and this indicates a possible explanation for mitochondrial dysfunction in the pathogenesis of IPF [55].

### 3.6. ROS and Stiffness

There are several forms of ROS production both in vitro and in vivo that include (1) electron leakage from mitochondria and resulting superoxide production, (2) enzymatic production during the oxidation and peroxidation reactions, and (3) released from leukocytes in immune reactions [56]. A systematic review of oxidative stress biomarker levels in IPF patients indicated that their levels were significantly higher compared to control groups in several types of specimens (blood, plasma, serum, urine, sputum, BALF, and lung tissue) [30]. Therefore, ROS are concerning seeing as they can be one of the triggers of lung fibrosis. They initiate the migration of fibroblasts to injured areas and cause the transition of fibroblasts to myofibroblasts, which is crucial in IPF onset [57]. ROS cause direct activation of the RhoA-ROCK pathway that regulates ECM production including *α*-smooth actin (*α*-sma) and collagen I [58]. The disintegration of microtubules reduces basal respiration and energy consumption [46]. ROS affect integrin signal transduction because they decrease the activation of the Akt protein and reduce the actin polymerization more in response to stretching than in response to the attachment [59]. Based on the drosophila model of wound healing, ROS formation induces mitochondrial fragmentation and caspase activation via the ROCK pathway. High levels of ROS prompt high tissue tension through cell delamination and increased contractility. Biomechanical tissue changes are caused by high interfacial tension through circumpherical myosin assembly and activity [60]. Moreover, cyclic mechanical tensions also increase ROS production in cells through an actin cytoskeleton-dependent manner [61]. Cultures of pulmonary epithelial cells, in conditions simulating overdistension, suggest that mechanical ventilation may lead to mitochondrial ROS-induced lung injury [62]. Abnormal mitochondrial dynamics and morphology in the diaphragm confirm this theory in vivo [63].

### 3.7. ECM and Mitochondrial-Induced Apoptosis

In most cells, apoptosis is dependent on the mitochondrial pathway because cytochrome c and/or SMAC/Diablo released from mitochondria to the cytosol activate the caspase proteases. Permeabilization of OMM is controlled by the Bcl-2 family of proteins. Antiapoptotic members of the Bcl-2 family proteins (Bcl-2, Bcl-XL, Mcl-1) promote cell survival. Contrary, the proapoptotic (Bax and Bak, Bid, Bim, and Bad) members which induce apoptosis through recruitment of proapoptotic proteins and increased permeabilization of OMM [64]. Mitochondria are mechanosensitive, and the cytoskeleton transduces tension from the ECM on the mitochondrial network. In response, mitochondrial membrane potential decreases. The proapoptotic proteins contribute to stretch-induced mitochondrial apoptosis. Loss of the ECM attachment in epithelial cells leads to rapid recruitment of Bax. Moreover, F-actin rearrangements are necessary for mitochondrial clustering and activation of death receptors [65]. Colocalization of the BAX and Drp1 proteins indicates the association between fission and apoptosis [18]. Drp1 knock-down delays cytochrome release and preserves it from immediately activating apoptosis [65]. On the contrary, increased contact of fibroblasts with the ECM as well as TGF-*β* stimulation promotes cell survival via FAK signaling [66]. Integrins and TGF-*β* activated EGFR result in Erk activation and suppression of Bim. Therefore, they prevent Bim-mediated apoptosis. TGF-*β* has recently been indicated to stimulate EGFR and integrins via FAK and SFK to promote fibroblasts survival [64].

### 3.8. Calcium

The equilibrium and distribution of calcium in tissue segments are involved in MQC. Mitochondrial calcium levels are higher in IPF macrophages than in healthy subjects. It is caused by increased expression of the mitochondrial calcium uniporter (MCU) in response to ER stress and calcium release [67]. Calcium influx into the mitochondria impairs mitochondrial function [46]. An elevated calcium level causes loss of mitochondrial membrane potential, increased ROS emission, and affects PGC-1*α* expression [67]. Mitochondrial calcium signaling can also drive myofibroblast differentiation and fibrosis foci [68]. Calcium sensitive receptors are upregulated in activated lung fibroblasts that calcilytics reduce markers implicated in pulmonary fibrosis [69].

### 3.9. Cytokines

A great abundance of cytokines is released during inflammatory processes in the lung tissue of IPF patients. They were theoretically divided into several groups according to their activity: (1) tyrosine kinase and serine–threonine kinases: platelet-derived growth factor (PDGF), transforming growth factor-beta 1 (TGF-*β*1), vascular endothelial growth factor (VEGF), and fibroblast growth factor 21 (FGF-21); 2) G-Protein–coupled activation lysophosphatidic acid (LPA); (3) chemokines: tumor necrosis factor-alpha (TNF-*α*), chemokine C-C motif ligand 2 (CCL2, also called monocyte chemoattractant protein-1 [MCP-1]), and C-X-C motif chemokine 12 (CXCL12); and (4) other mechanisms: lysyl oxidase-like 2 (LOXL2), plasminogen activator inhibitors 1 and 2 (PAI-1; PAI-2), and tissue inhibitor of metalloproteinases (TIMP) [1, 8, 9, 11, 70]. An association between mitochondrial dysfunction and cytokine production has been established in several studies [71–73]. Moreover, cytokines produced in one type of cell regulate several cellular pathways in another [26]. Paracrine activation of fibroblasts regulates senescence-associated secretory phenotypes (SASP) and mitochondrial equilibrium [8].

#### 3.9.1. Influence on Mitochondria

TGF-*β*, released mainly from alveolar epithelial cells type II and macrophages, affects the mitochondrial balance in local cells through (1) decreasing mitophagy regulators such as PINK1, Parkin, and SIRT3 [74, 75]; (2) diminishing mitochondrial transmembrane potential regulating ETC function, especially at complex IV, resulting in increased byproduct production of mtROS and lactate cell contents [26]; and (3) upregulating mitochondrial mass in fibroblasts acting via STAT3 and SMAD2/3-C/EBPb-PRMT1 signaling pathways [76] (Figure 1.  Subsection 1).

G-coupled receptor kinases (GRK) and B-arrestins constitute important regulators of mitochondrial function [77]. They are involved in mitochondrial-dependent cell death regulating mitochondrial cytochrome *c* release and downstream caspase activation [78]. It was proven that the overexpression of GRK2 results in increased mitochondrial mass and enhanced oxidative respiration [77].

#### 3.9.2. Influence on ECM Stiffness

TGF-*β* is the most studied fibrotic process stimulator [76]. TGF-*β* induces fibrosis in mouse models of IPF. Moreover, an increased level of the TGF-*β* expression is considered a profibrotic mediator in IPF patients [26]. This cytokine produced by epithelial cells, eosinophils, macrophages, and fibroblasts is involved in airway wall remodeling. TGF-*β* also induces differentiation of fibroblasts to myofibroblasts. Myofibroblasts deposit an excessive amount of ECM components. The relevance of mechanical stresses with the TGF-*β* pathway coactivation is well known and crucial for disease progression [13].

G protein-coupled receptors (GPCRs) participate in mechanotransduction. GPCRs can be activated directly via stimulation by mechanical forces and indirectly via conformational changes consistent with *β*2-adrenergic receptor activation [79].

Based on in vivo and in vitro studies, crosstalk between the ECM and mitochondria is summarized in Table 1.

## 4. Senescence

Senescence is a biological hallmark in terms of cell ageing. In this condition, cells are in a state of stable replicative arrest. Cellular senescence is a pathological feature of IPF lungs and is evident in experimental lung fibrosis models [89]. Senescence affects both fibroblasts and alveolar epithelial cells in IPF patients. Indeed, senescence contributes to age-related respiratory diseases including IPF [90]. Senescence is induced by premature ageing, DNA damage, oxidative stress, telomere shortening, oncogene activation, and mitochondrial dysfunction [91–94]. Mitochondria play a special role in senescence through mtROS production, bioenergy imbalance, and p53/p21 and/or p16/pRb pathway signal triggering [95]. The outcome of the mentioned processes is organ ageing which results in inflammation, loss of tissue regeneration, and fibrosis. Moreover, senescent cells acquire a senescence-associated secretory phenotype (SASP) which includes a broad spectrum of growth factors, cytokines, and chemokines. This serves to mediate paracrine local tissue inflammation and promote tissue degeneration [92]. SASP has a direct influence on mitochondrial mass, abnormal morphology, biogenesis declines, and insufficient mitophagy [22, 94]. Additional mechanical stress is a cofactor that accelerates disease progression and lung remodeling in a senescent mechanism [93].

**Figure 2:**
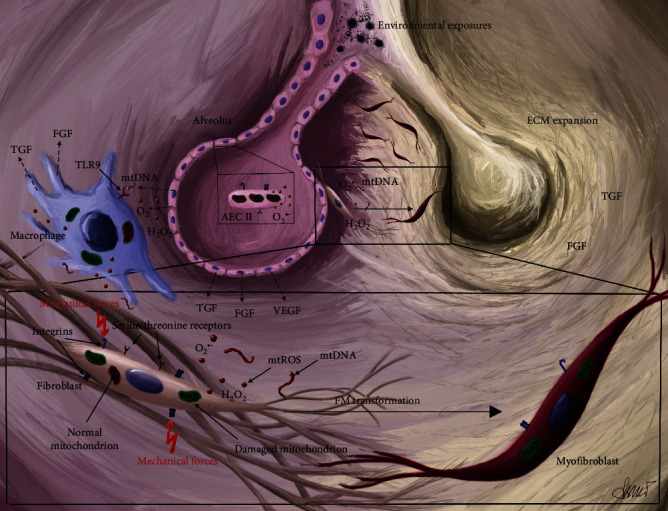
Selected aspects of the role of mitochondria in IPF pathophysiology. Exposure to harmful environmental factors, including tobacco smoke, dust, silica, farming, infections drugs, gastroesophageal reflux microbial, and viral agents induces mitochondrial dysfunction in airway epithelial cells (AECs cells). Damaged mitochondria (green) release mtROS (red dots) and mtDNA (red wavy lines) to the microenvironment. AECs have undergone the transition to senescent epithelial cells with SASP and secrete cytokines (TGF, FGF, VEGF, etc.). Mentioned factors initiate the inflammatory process with macrophage enhancement (blue spherical cells). Local fibroblasts exposed to harmful conditions transform to myofibroblasts (FM transformation). Myofibroblasts are the main cells responsible for ECM protein production in IPF pathobiology. The interstitial area becoming abundant in collagen I periostin 1, osteopontin, fibronectin, and fibulin. Increased tensions, mtROS, mtDNA, and cytokines maintain inflammatory reaction and gradually cause AECs apoptosis. Scarification of local lung tissue and alveoli degeneration precedes lung fibrosis.

Selected aspects of the role of mitochondria in IPF pathophysiology are summarized in Figure 2.

## 5. Clinical Implications

### 5.1. IPF Treatment Mitigates the Progression of Fibrosis via Mitochondria

Currently, two types of therapies were approved in IPF treatment, nintedanib, and pirfenidone. Both antifibrotic agents inhibit the decline of FVC, prevent acute exacerbations, and slow disease progression [5]. In half of the cases, the exacerbation is involved in patient death. Acceptable management of disease exacerbation includes corticosteroids and immunosuppressants [96].

Pirfenidone is an antifibrotic agent with an unknown mechanism of action. Studies conducted on animals and cell cultures have revealed that pirfenidone inhibits fibroblast proliferation and collagen production, reduces the activity of TK/S-TK, and decreases cellular markers of fibrosis [96]. Pirfenidone has protective properties on mitochondrial membranes. Pirfenidone stabilizes MMP that maintains ATP production. Therefore, pirfenidone inhibits free radical production and improves mtDNA by stabilizing the number of its copies [97]. In IPF patients, pirfenidone inhibits the secretion of serum mtDNA which is an inductor of fibroblasts to myofibroblasts differentiation [67]. Pirfenidone induces PARK2-mediated mitophagy. In mitophagy-driven fibrosis, pirfenidone reduces lung fibrosis in animal models of IPF. This presented hypothesis explains the antibiotic properties of pirfenidone and its potential mechanism of action in IPF patients [98]. Partially, this theory was confirmed by a USP30 or STAT3 inhibition in the study on in vivo bleomycin-induced lung fibrosis models. USP30 inhibitors increasing mitophagy acts with comparable efficiency to pirfenidone in the reduction of hypercellularity and collagen production [27, 55].

Nintedanib is a TK/S-TK inhibitor. Based on bleomycin-induced IPF animal models, nintedanib mitigates lung inflammation and reduces lung fibrosis. Nintedanib inhibits cell migration, TGF-*β*-induced collagen deposition, and fibroblast to myofibroblast differentiation [10].

Latent TGF-*β* cytokines have to be transformed into their active form via *α*v*β*6 integrin. Inhibition of this process was utilized in a designed monoclonal antibody against *α*v*β*6. TGF-*β* is also involved in mitochondrial regulation, and this was underlined in the section, “Cytokines” [80].

Contrary to TGF-*β*, the expression of another cytokine, FGF-21, attenuates pulmonary fibrogenesis through mitigating oxidative stress in vivo and in vitro conditions [99].

FGF-21 attenuates pulmonary and hepatic fibrosis [100]. Several studies on bleomycin-induced models of IPF confirm that FGF-21 decreases deposition of the ECM, limits the intense infiltration of inflammatory cells, and slows remodeling of tissue architecture in the lungs [99]. Lipid peroxidation (MDA) assay, the activity of superoxide dismutase (SOD), and total antioxidant capacity (T-AOC) indicate that FGF-21 inhibits oxidative stress. Therefore, the increased expression of FGF-21 was associated with impairment of mitochondrial oxidation.

Other compounds which have an influence on mitochondria and could be potential therapeutic agents for IPF therapy are PGC-1*α*, sirtuin 3 [101], MitoQ [102], and SASP inhibitors [103] (summarized in Table 2). The common feature of these compounds is that they decrease lung fibrosis developed in the mechanism of mitochondrial dysfunction. Therefore, the role of mitochondria, oxidative stress, and ECM properties including tension and composition has to be further investigated.

## 6. Conclusions

Numerous aspects of mitochondrial pathology including biogenesis dysfunction, fission-fusion equilibrium, mitophagy impairment, or mtROS overproduction seem to play a role in the pathobiology of lung fibrosis. In addition, an increasing body of evidence suggests considering crosstalk between the ECM and mitochondria in this context.

Moreover, mitochondria and integrins seem to be important players in antifibrotic treatment. Further research on mitochondrial functioning in IPF may potentially lead to a deeper understanding of its pathobiology and aid in elaborating new therapeutic approaches, which can ameliorate prognoses.

## Figures and Tables

**Table 1:** Summary of interactions between the ECM and mitochondria in pulmonary fibrosis in vivo and in vitro studies.

Interaction with mitochondria via	In vivo studies	In vitro studies	Ref.
Mechanical tensions	(i) The lung tissue biomechanic alteration influencing cells via intracellular organelle dysfunction includes mitochondria	(i) Mechanical stress influences on mitochondria structure and function, reduces mitochondrial membrane potential and ATP production, and induces ROS production, alter fusion, and fission	[18, 37–39, 63]
Cytoskeleton		(i) Cytoskeletal toxins induce shortening of mitochondria	[46]
Integrins	(i) *α*V*β*6-mediated activation of TGF-*β* is necessary for the development of fibrosis in lung-disease models	(i) Integrin ligands stimulate mitochondrial function	[50, 51, 53, 80]
Signaling	(i) Low PTEN levels induce fibrogenesis	(i) FAK and STAT3 inhibition abolished mitochondrial function	[52–54, 81]
ROS	(i) Mitochondrial ROS in AEC mediate mtDNA damage and fibrosis (ii) Mitochondrial ROS are essential to the development of pulmonary fibrosis (iii) Antioxidant treatment attenuates the bleomycin-induced oxidative burden and subsequent pulmonary fibrosis (iv) The absence of extracellular superoxide dismutase exacerbates conditions that lead to inflammation and pulmonary fibrosis	(i) mtDNA leads to ROS production, inflammation, and I consequence fibrosis (ii) TGF-*β*1 induces prolonged mitochondrial ROS generation	[73, 82–85]
Apoptosis	(i) Low PTEN levels inhibit mitochondrial-dependent apoptosis	(i) The proapoptotic proteins contribute to stretch-induced mitochondrial apoptosis	[52, 65, 81]
Calcium	(i) The S100 calcium-binding protein A4 level is elevated in the lungs of patients with IPF	(i) Calcium influx into the mitochondria impairs mitochondrial function (ii) Calcium-sensitive receptors are upregulated in activated lung fibroblasts and reduce markers implicated in pulmonary fibrosis	[46, 69, 86]
Cytokines	(i) TGF-*β* induces ECM and ROS overproduction, decreases mitochondrial membrane potential, and inhibits mitophagy	(i) Mitochondria dysfunction increase cytokines production in lung epithelium	[73, 87, 88]

**Table 2:** Properties of potential mitochondrial therapeutic agents in IPF treatment.

Potential therapeutic agents	Mechanism of action	Advantages	Disadvantages	Ref.
USP30 inhibitors	Mitophagy stimulation	Utilization of dysfunctioning of mitochondria	Excessive elimination of mitochondria	[26–28]
FGF-21 analogues	Oxidative stress inhibition	Decease ROS and ECM production	Unpredictable metabolic effects	[99, 104]
MitoQ	Increase mitochondrial respiration efficiency	Inhibition of oxidative stress, ROS production, and mitochondria dysfunction	High therapeutic dose	[102]
Sirtuin 3	Mitochondrial deacetylation level regulation	Inflammation suppression Inhibition of oxidative stress Apoptosis regulation Autophagy regulation	Hiperautophagy Apoptosis inhibition and cancerogenesis	[101]
STAT3 inhibitors	Induction of mitophagy	Utilization of malfunctioning mitochondria	Excessive elimination of mitochondria	[55]
Integrin's blockers	Inhibition of latent TGF-*β* activation and mechanical stress transmission	Mitigate mechanical stress and TGF-*β* induced lung fibrosis	Crossactivity on inhibition integrin-ligand binding, integrin-mediated cell adhesion, and TGF-*β* signaling	[105]
SASP inhibitors	Inhibition of senescence-associated secretory phenotype (SASP) transition	The broad spectrum of antifibrotic action	Molecular targets with unpredictable effect	[75, 103]
PGC-1*α* analogs	Mitochondrial biogenesis stimulation	Increase mitochondria population	Increase ROS production	[28, 106]
